# Simian iPS-derived hepatocytes support *Plasmodium cynomolgi* infection and hypnozoite formation

**DOI:** 10.3389/fcimb.2026.1871289

**Published:** 2026-08-14

**Authors:** Mélanie Pellisson, Anne-Marie Zeeman, Thierry Doll, Lucy Kirchhofer-Allan, Sven Schuierer, Guglielmo Roma, Erika L. Flannery, Sebastian Mikolajczak, Clemens H. M. Kocken, Pascal Mäser, Matthias Rottmann, Matthias Müller

**Affiliations:** 1 Swiss Tropical and Public Health Institute (Swiss TPH), Allschwil, Switzerland; 2 University of Basel, Basel, Switzerland; 3 Novartis Institutes for Biomedical Research, Novartis Pharma AG, Basel, Switzerland; 4 Department of Parasitology, Biomedical Primate Research Centre, Rijswijk, Netherlands; 5 Novartis Institute for Tropical Diseases, Novartis Pharma AG, Emeryville, CA, United States

**Keywords:** hypnozoite, iPS-derived hepatocytes, malaria, *Plasmodium cynomolgi*, *Plasmodium vivax*

## Abstract

**Introduction:**

*Plasmodium cynomolgi* is one of the few malaria parasite species that forms dormant liver stages known as hypnozoites and is therefore a suitable model for *Plasmodium vivax*, the causative agent of recurrent malaria. Very little is known about the biology and pharmacology of liver stage dormancy, which hampers the search for compounds with anti-hypnozoite activity.

**Methods:**

We present the development of a *P. cynomolgi in vitro* infection model using stem cell-derived hepatocyte-like cells from *Macaca fascicularis.* iPS cells were established on feeder free condition and differentiated into hepatocyte-like cells via inducible overexpression of key transcription factors.

**Results:**

The generated cells were infected with *P. cynomolgi* sporozoites, and hypnozoite formation as well as schizont development were confirmed by immunofluorescence microscopy.

**Discussion:**

This simian iPS-derived model is a promising alternative to current *P. vivax in vitro* infection systems to study the mechanisms underlying liver stage dormancy.

## 1 Introduction

In 2024, malaria had an estimated global incidence of 282 million and a death toll of 610 000 (WHO, 2025). Thus, despite the recent successes in the fight against malaria (Rabinovich et al., 2017), the disease remains a main killer. The majority of deaths are due to *Plasmodium falciparum*, the causative agent of malaria tropica. *Plasmodium vivax* has a wider global distribution and causes relapsing malaria, which is less fatal than malaria tropica but nevertheless imposes a substantial disease burden, particularly in Asia and South-America (Angrisano and Robinson, 2022; Weiss et al., 2025). Malaria being anthroponotic, elimination and even eradication are theoretically possible. However, in addition to drug-resistant *Plasmodium* parasites and insecticide-resistant *Anopheles* mosquitoes (Poespoprodjo et al., 2023), there is an ultimate hindrance to malaria eradication: the hypnozoites of *P. vivax* (Adekunle et al., 2015; Robinson et al., 2015; Commons et al., 2020; Angrisano and Robinson, 2022). Hypnozoites are small, uninucleated, intracellular stages that stay dormant for months or even years in the liver of infected carriers (Krotoski et al., 1980, 1982; Dembele et al., 2014), building a hidden reservoir of parasites that, upon reactivation, causes relapsing malaria. Primaquine and tafenoquine are the only drugs that eliminate hypnozoites (Llanos-Cuentas et al., 2019), but their use is limited due to severe side effects in glucose-6-phosphate dehydrogenase-deficient patients (a common genetic trait in malaria endemic countries) (Alving et al., 1960; Chu et al., 2017; Rueangweerayut et al., 2017). Therefore, new molecules with anti-hypnozoite activity are needed.

Although recent transcriptomic analyses at single cell level have provided some hints on the mechanisms by which *Plasmodium* alters the host cell machinery to survive (Voorberg-van der Wel et al., 2017; Bertschi et al., 2018; Mancio-Silva et al., 2022; Ruberto et al., 2022), the hypnozoite biology and the mechanisms underlying dormancy and reactivation are still poorly understood. This lack of knowledge can be explained by the difficulties linked to both the cellular system used for infection assays and the *in vitro* work with *Plasmodium vivax.* Most studies on *P. vivax* hypnozoites have been conducted with human primary hepatocytes as host cells (Mazier et al., 1984; Gural et al., 2018; Roth et al., 2018; Chua et al., 2019a; Vantaux et al., 2022). These, however, present problems regarding *in vitro* maintenance, donor-to-donor variability, and ethical considerations. To overcome these challenges, hepatocytes derived from human induced pluripotent stem (iPS) cells have been used as an alternative in both 2D and 3D systems and were shown to be permissive to *P. vivax* and *P. falciparum* (Ng et al., 2015; Subramani et al., 2020; Yang et al., 2023; Nitaramorn et al., 2024). Additional hurdles encountered when working with *P. vivax* is the absence of long-term *in vitro* culture system and the restricted sporozoite sourcing to endemic areas (Gunalan et al., 2020). The best surrogate organism for *P. vivax* is *P. cynomolgi*, a closely related and more accessible malaria parasite of non-human primates whose blood-stages can be cultured (Chua et al., 2019b) and which also produces hypnozoites (Krotoski et al., 1980). Here we present for the first time a solution that overcomes the limitations of both *P. vivax in vitro* work and primary host cell usage by establishing an *in vitro* system for *P. cynomolgi* hypnozoites that uses cynomolgus monkey hepatocyte-like cells generated by iPS cell technology.

## 2 Methods

### 2.1 Primary cells

Cynomolgus monkey fibroblasts used for iPS cell generation were obtained from ZenBio (monkey PDF091812) and Novartis (monkey 5501). Cynomolgus monkey primary hepatocytes were purchased from Biopredic. Human dermal fibroblasts used for iPS and hepatocyte generation were purchased from Invitrogen and human primary hepatocytes from KaLy-Cell. The donors provided written informed consents.

### 2.2 Cynomolgus monkey iPS cell generation

Primary cynomolgus monkey dermal fibroblasts were reprogrammed using Sendai viruses of CytoTune-iPS 2.0 Sendai Reprogramming Kit (Life Technologies, A16517) according to the standard protocol. Cells and viruses were plated on irradiated mouse embryonic fibroblasts (Merck Millipore, PMEF-CF1) and cultivated with human embryonic stem cell (hESC) medium [KnockOut DMEM; 4.5 g/L glucose, 0.11 g/L sodium pyruvate, without glutamine (Gibco, 10829018), 20% KnockOut Serum Replacement (Gibco, 10828028), 1% glutaMAX (Gibco, 35050038), 1% MEM Non-Essential Amino Acids (NEAA) (Gibco, 11140035), 1% penicillin/streptomycin (Gibco, 15140-122), 10 ng/mL human basic fibroblast growth factor (bFGF) (Invitrogen, 13256029), and 0.1% β-mercaptoethanol (Gibco, 31350010)]. Colonies with hallmark of pluripotent morphology were readily visible between 2–3 weeks after transduction. These pluripotent colonies were picked and subcloned multiple times either on feeder cells or on Matrigel (BD Biosciences, 354277) coated plates in hESC or mTeSR1 medium (Stem Cell Technologies, 05850). This serial subcloning was performed until remnants of Sendai virus RNA were no longer detected and the morphology looked stable. As constant bFGF activity is essential for cynIPS maintenance, mTeSR1 medium was progressively replaced by StemFlex medium (Gibco, A3349401) which allows maintenance of bFGF levels over time. Pluripotency was assessed by immunofluorescence staining, fluorescence-activated cell sorting (FACS) analysis, and differentiation potential in all three germ layers (see sections below). Karyotype of each selected line was checked by array Comparative Genomic Hybridization (aCGH method) (Cell Line Genetics) and all showed a normal karyotype.

### 2.3 Flow cytometry analysis for bFGF experiment and iPS characterization

CynIPS cells were dissociated with TryplE (Invitrogen, 12604), followed by fixation and permeabilization using Cytofix/Cytoperm reagent (BD Biosciences, 554655 and 51-20S1KZ) according to the manufacturer. Cells were stained with Nanog-PE (BD Biosciences, 560589a), Oct3/4-PerCP (BD Biosciences, 560794) and Sox2-A647 (BD Biosciences, 562139) intracellular primary antibodies for 45 min at 4 °C, washed twice by sedimentation at 1000 rpm followed by resuspension in ice cold Cytoperm reagent, and finally resuspended in 1% fetal calf serum (FCS) in phosphate buffered saline (PBS) (Invitrogen, 14190). Cells were subsequently analyzed on a CyAn flow cytometer (Beckmann Coulter).

### 2.4 Karyotyping

For each tested cell line, 1.5E05 cells were seeded onto a T25 Matrigel coated flask in StemFlex medium supplemented with 10 µM rock inhibitor (Merck Millipore, Y-27632). Medium was refreshed the following day without rock inhibitor and lived cells were sent over to Cell Line Genetics when 20-30% confluence was reached. Karyotype of each selected line was checked by aCGH method.

### 2.5 Differentiation potential

#### 2.5.1 Endoderm differentiation

Endodermal cells were induced from cynIPS cells using STEMdiff Definitive Endoderm (DE) Kit (Stem Cell Technologies, 05110). CynIPS cells were seeded onto a 24-well plate (TPP, 92024) coated with Matrigel and cultivated with StemFlex medium until culture reached 95-100% confluence. Cells were washed by sedimentation at 1000 rpm followed by resuspension in PBS, and medium was switched to STEMdiff medium with 1% DE supplements A and B. The next day, medium was switched to STEMdiff medium with 1% DE supplement B. Medium was changed every day. Cells were fixed after 5 days of differentiation with 4% paraformaldehyde (Electron Microscopy Sciences, 15714) in PBS and immunofluorescence staining was performed with forkhead box A2 (FoxA2) (1:50 dilution, Abcam, ab60721) and Oct4 (1:1,000 dilution, Stemgent, 09-0023) primary antibodies.

#### 2.5.2 Ectoderm differentiation

Neuronal precursors were differentiated from cynIPS cells using a modified dual Smad inhibition protocol. 6E04 undifferentiated iPS cells were seeded onto a 96-well ultra-low attachment (ULA) plate (Costar, 7007) in 0.1 mL StemFlex medium with 10 ng/mL penicillin/streptomycin and 10 μM rock inhibitor to prevent apoptosis. 24 hours after seeding, Embryoid Bodies (EBs) were formed and 0.1 mL fresh StemFlex medium were added to the wells. The next day, 30 EBs were transferred to a 4-well Matrigel coated plate with StemFlex medium (7 or 8 EBs seeded per well). 6 hours later, StemFlex medium was replaced by neural induction medium (20% KnockOut™ Serum Replacement, 0.1 mM MEM NEAA, 0.1 mM β-mercaptoethanol, 75% DMEM/F12/glutaMAX; 1% penicillin/streptomycin, 10 ng/mL human bFGF, 10 μM SB431542 (Tocris, 1614), and 1 μM LDN193189 (Stemgent, 04-0074). Medium was changed every other day until day 13. Cells were fixed after 14 days of differentiation with 4% paraformaldehyde in PBS for 10 min at room temperature and immunofluorescence staining was performed with βIII-tubulin (1:5,000 dilution, Sigma, T8660), 200 kDa neurofilament (NF200) (1:6,000 dilution, Abcam, ab72996), neuronal nuclei (NeuN) (1:500 dilution, Merck Millipore, ABN78), and Oct4 (1:1,000 dilution, Stemgent, 09-0023) primary antibodies.

#### 2.5.3 Mesoderm differentiation

6E04 undifferentiated cynIPS cells were seeded onto a 96-well ULA plate in 0.1 mL StemFlex medium with 10 ng/mL penicillin/streptomycin and 10 μM rock inhibitor to prevent apoptosis. 24 hours after seeding, EBs were formed and 0.1 mL fresh StemFlex medium were added to the wells. The next day, medium was replaced by STEMdiff Mesoderm Induction Medium (Stem Cell Technologies, 05220). Medium was refreshed daily until spontaneous EB contraction was observed after 10 days of differentiation.

### 2.6 Generation of cynomolgus monkey hepatocyte-like cells

CynIPS cells were harvested and seeded (1.1E05 cells/well) onto a 96-well plate (Greiner, 655090) coated with Laminin 521 (Biolamina, LN521) in StemFlex medium supplemented with 100 µg/mL geneticin (G418) (Gibco, 10131035), and 10 µM rock inhibitor. The next day, cells were washed by sedimentation at 1000 rpm followed by resuspension in PBS, and differentiation was started (day 0) in Chemically Defined Medium (CDM) [50% IMDM (Invitrogen, 31980030), 50% DMEM/F12 (HAM) (Invitrogen, A14625DJ), 1% Insulin-Transferrin-Selenium (ITS) (Invitrogen, 51500056), 0.1% CD Lipid Concentrate (Invitrogen, 11905031) and 2% bovine serum albumin (BSA) (Sigma, A7979)] supplemented with 1 µg/mL doxycycline (Dox). The next day, medium was changed with CDM supplemented with 1 µg/mL Dox. On day 2, medium was switched to William’s E medium (no phenol red, Gibco, A1217601) with Primary Hepatocyte Maintenance Supplements (Gibco, CM4000) and 1 µg/mL Dox. Medium was refreshed the next day. On day 4, Dox induction was stopped and William’s E medium containing Primary Hepatocyte Maintenance Supplements was supplemented with 5 µM of a combination of compounds (doramapimod, dihydroorotate dehydrogenase (DHODH) inhibitor, and rapidly accelerated fibrosarcoma (Raf) inhibitor) to maintain cynHLCs until 17 days of differentiation. Medium was refreshed daily.

### 2.7 Generation of human hepatocyte-like cells

Human iPS cells were harvested and seeded onto a 96-well Laminin 521 coated plate (8E04 cells/well) in mTeSR1 medium supplemented with 100 µg/mL G418 and 10 µM rock inhibitor. The next day (day 0), cells were washed by sedimentation at 1000 rpm followed by resuspension in PBS, and differentiated for 3 days in CDM supplemented with 1 µg/mL Dox (medium was refreshed daily). On day 3, medium was switched to William’s E medium with Primary Hepatocyte Maintenance Supplements and 1 µg/mL Dox. Medium was refreshed on day 4 and 5. On day 6, Dox induction was stopped, and cells were further differentiated in William’s E medium containing Primary Hepatocyte Maintenance Supplements until day 25. Medium was refreshed every other day.

### 2.8 Immunofluorescence analysis

Cells were fixed with 4% paraformaldehyde in PBS, permeabilized with Triton X-100 (Sigma, T8787) in PBS and stained with primary antibodies visualized with appropriate fluorescently labeled secondary antibodies (Alexa Fluor^®^). Cultures were counterstained with the nuclear marker Hoechst (Invitrogen, 33342) and immunostained samples were imaged using a Zeiss LSM 700 microscope. Primary and secondary antibodies references can be found in **Supplementary Table 1**.

### 2.9 Periodic acid schiff staining (Sigma, 395-1KT)

Fixed samples were rinsed with deionized water and then placed in 0.5% periodic acid aqueous solution for 5 min at room temperature. Samples were then rinsed with running tap water for 3 min and quickly rinsed with deionized water before being placed in Schiff reagent solution for 15 min. The same rinsing steps as described above were used and samples were then counterstained with hematoxylin for 2 min. Samples were rinsed with deionized water for 3 min. Samples were rinsed twice with 70% ethanol, twice with 96% ethanol and twice with 100% ethanol for 1 min each. Samples were covered with PBS before imaging.

### 2.10 Oil red O staining

Fixed samples on microscope slides were gently rinsed twice with deionized water and placed in 60% isopropanol for 5 min. 30 mL Oil Red O stock solution (60 mg Oil Red O (BioVision, K580-24-3) in 20 mL 100% isopropanol) was mixed with 20 mL deionized water and filtered using a 0.2 µm syringe filter to make Oil Red O working solution. Samples were incubated with working solution for 15 min and then rinsed two to five times with deionized water until excess stain was no longer apparent. Samples were counterstained with hematoxylin (BioVision, K580-24-2) for 1 min. Samples were rinsed two to five times with deionized water before imaging.

### 2.11 Enzyme-linked immunosorbent assay

To assess albumin secretion capacity of hepatocytes, cynIPS cells were harvested and seeded onto a 24-well Laminin 521 coated plate (7.5E05 cells/well) and differentiated as previously described. Culture medium was collected every 24 hours before the next medium change. ELISA assay was performed with the collected supernatants according to the provider standard protocol (Immunology Consultants Laboratory, E-80AL).

### 2.12 Hepatocyte survival screen

CynIPS cells were harvested and 2.5E04 cells/well were seeded using a multidrop dispenser onto 384-well Laminin 521 coated plates (Corning, 3712) in StemFlex medium supplemented with 100 µg/mL G418, and 10 µM rock inhibitor. Cells were differentiated as described above until day 4. At day 4, Dox induction was stopped, medium was replaced by William’s E medium with Primary Hepatocyte Maintenance Supplements and cells were treated with the Novartis compound library in the Echo^®^ Liquid Handling platform (Labcyte Inc.). Cells were returned to overnight incubation at 37 °C, 5% CO_2_. Cells were treated with the Novartis compound library every other day after medium change. At day 13, bright field images of all plates were acquired with a high-throughput high-content imaging system (Operetta, PerkinElmer) and CellTiter-Glo luminescent cell viability assay (Promega, G7570) was performed. Luminescent readings were obtained using an EnVision multilabel plate reader (PerkinElmer) after 10 min incubation at room temperature. Generated data were analyzed in TIBCO Spotfire^®^ (TIBCO Software Inc.).

### 2.13 RNA sequencing

Total RNA was isolated using the Direct-zol RNA MiniPrep Kit (Zymo Research, R2071) including on-column deoxyribonuclease (DNase) digestion according to the manufacturer’s instructions. RNA sequencing libraries were prepared using the Illumina TruSeq RNA Sample Prep kit v2 (Illumina, RS-122-2001) and sequenced using the Illumina HiSeq2500 platform. Samples were sequenced to a length of 2 × 76 base pairs. Read pairs from cynomolgus iPS cells, HLCs, and primary hepatocytes were mapped to the *Macaca fascicularis* genome and the cynomolgus gene transcripts from Refseq by using an in-house gene quantification pipeline (Schuierer and Roma, 2016). The human genome (hg38) was used for the mapping of human HLCs, primary hepatocytes, and liver related cancer cell lines. Genome and transcript alignments were used to calculate gene counts based on Ensembl gene IDs. The corresponding BAM (Binary Alignment Map) files are available via the European Nucleotide Archive.

### 2.14 Generation of *P. cynomolgi* sporozoites

The Biomedical Primate Research Centre (BPRC) is an AAALAC-certified institute. All rhesus macaques (*Macaca mulatta*) used in this study were captive bred for research purposes and were housed at the BPRC facilities in compliance with the Dutch law on animal experiments, European directive 2010/63/EU, and with the Standard for Human Care and Use of Laboratory Animals by Foreign institutions, identification number A5539-01, provided by the National Institutes of Health (NIH). Prior to the start of experiments, all protocols were approved by the local independent ethical committee, according to Dutch law. For each batch of infected mosquitoes, one rhesus macaque was infected with 1E06 *P. cynomolgi* M strain blood stage parasites and bled at peak parasitemia, after which the monkey was cured from the malaria infection by intramuscular injection of 7.5 mg/kg chloroquine on three consecutive days. Approximately 1,200 female *Anopheles stephensi* mosquitoes strain Sind-Kasur Nijmegen (Nijmegen University Medical Centre St. Radboud, Department of Medical Microbiology) were fed with this blood using an ex vivo glass feeder system.

### 2.15 Sporozoite inoculation of cynHLCs

Sporozoite inoculation of cynHLCs was performed at Novartis according to (Dembele et al., 2014). *P. cynomolgi* sporozoites were isolated at the BPRC and shipped to Novartis in Leibovitz L15 medium (Invitrogen, 11415-056) with 3% FCS and 2% penicillin/streptomycin at 4 °C to ensure good sporozoite infectiousness. Upon arrival, 4-days differentiated cynHLCs were infected with different sporozoite densities in 96-well plates. Cultures were kept at 37 °C in 5% CO_2_ with daily medium changes. To evaluate the development of *P. cynomolgi* liver stages, cultures were fixed with 4% paraformaldehyde at the indicated time points.

### 2.16 Immunofluorescence staining of liver stage parasites

Infected hepatocytes were fixed with 4% paraformaldehyde for 30 min, followed by overnight incubation at 4 °C with rabbit primary antibodies against *P. cynomolgi* 70 kilodalton heat shock proteins (Hsp70) and rat primary antibodies against *P. cynomolgi* upregulated in infective sporozoites gene 4 protein (UIS4) both diluted in 1% BSA and 0.3% Triton X-100 in PBS solution. Subsequently, donkey secondary immunoglobulin G (IgG) Alexa Fluor^®^ 555-conjugated anti-rabbit antibodies (1:1,000 dilution, Invitrogen, A31572), chicken secondary IgG Alexa Fluor^®^ 594-conjugated anti-rat antibodies (1:1,000 dilution, Invitrogen, A21471) were added for three hours at room temperature. Nuclei were counterstained with Hoechst for 10 min at room temperature, samples were finally covered with PBS and viewed under an inverted microscope (Leica DMI6000 or Zeiss LSM 700). Image acquisition and post processing were performed with proprietary Leica or Zeiss software, LAS X or Zen 2012 respectively. Primary and secondary antibodies references can be found in **Supplementary Table 1**.

### 2.17 Statistical analysis

**Figure 1:**
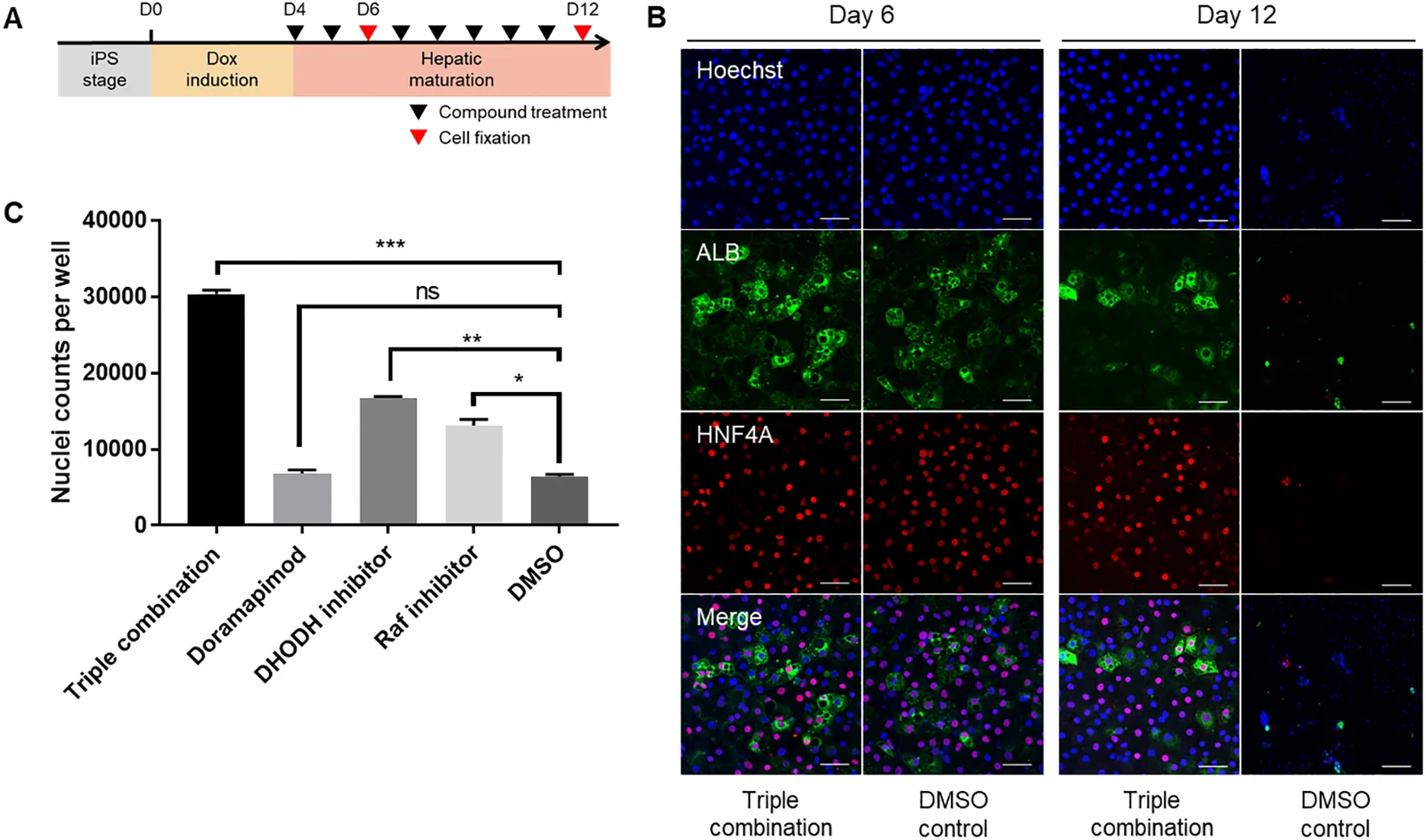
Survival screen allows identification of compounds that improve cynHLCs longevity. **(A)** Schematic representation of the experiment conducted to assess the effect of hit compounds identified to promote cynHLCs survival. Cells were treated daily from day 4 (D4) to day 12 (D12). **(B)** Immunofluorescence at day 6 and 12 of treated (triple combination) and untreated (DMSO control) cynHLCs with antibodies to ALB (green) and HNF4A (red). Nuclei were visualized with Hoechst (blue). Scale bars, 50 μm. **(C)** Bar chart representing the nuclei counts of treated and untreated cynHLCs at day 12. Error bars represent standard deviation. n=3 *, p<0.05; **, p<0.01; ***, p<0.001).

Data are presented as mean ± standard deviation (Stdev). P < 0.05 was considered statistically significant (ns, not significant (P > 0.05); ^∗^P < 0.05; ^∗∗^P < 0.01; ^∗∗∗^P < 0.001; ^∗∗∗∗^P < 0.0001). GraphPad Prism 7 (GraphPad Software) was used for statistical analyses. In **Figure 1C**, one-way analysis of variance (ANOVA) was used to compare the groups followed by Dunnett’s *post hoc* test.

## 3 Results

### 3.1 Generation and maintenance of iPS cells from *Macaca fascicularis*

**Figure 2:**
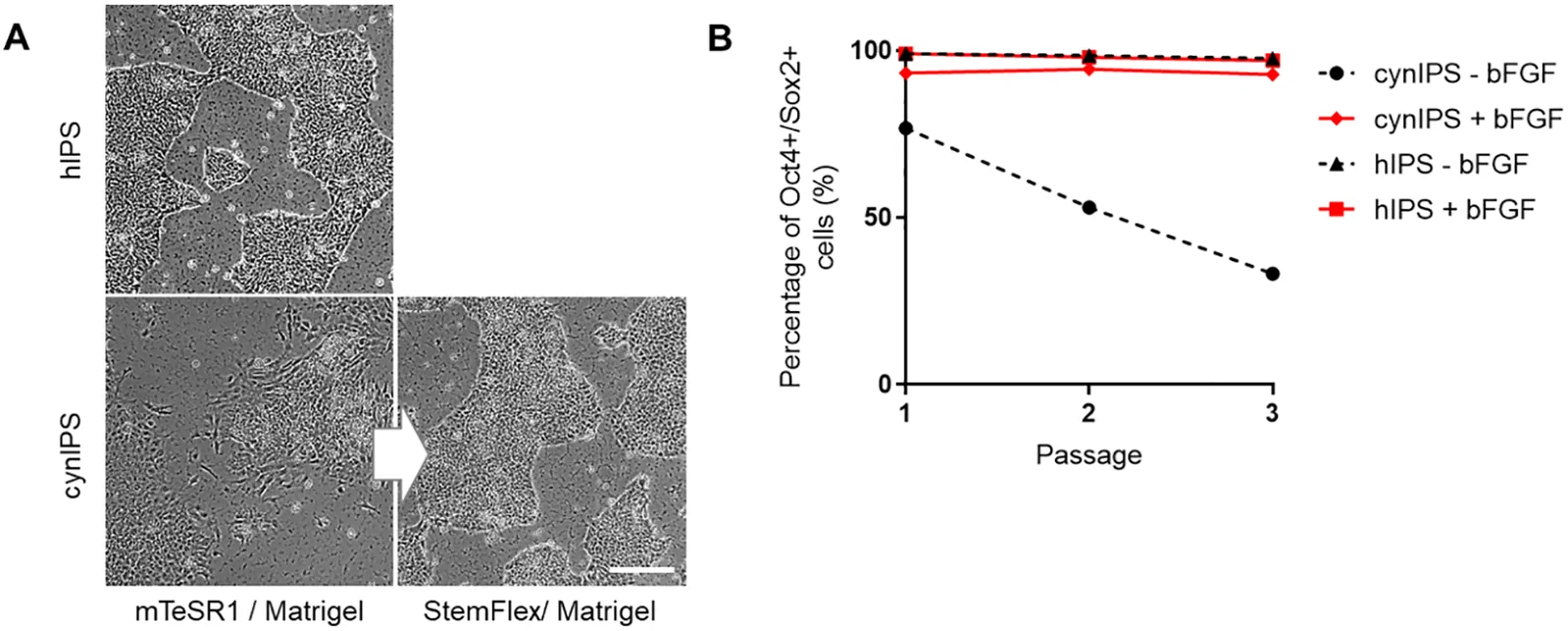
Cynomolgus vs. human iPS cell maintenance after generation. **(A)** Brightfield images of human (h) and cynomolgus (cyn) iPS cells. CynIPS spontaneously differentiated when cultivated with mTeSR1 medium. Switching to StemFlex medium with stabilized bFGF at 10 ng/mL was essential for cynIPS cell maintenance. Scale bar, 200 µm. **(B)** FACS analysis of human and cynomolgus iPS cells (cultivation +/- bFGF) with Oct4 and Sox2 pluripotent markers.

CynIPS cells were generated from dermal skin fibroblasts of two different cynomolgus macaques (*Macaca fascicularis*) by reprogramming, using Sendai viruses bearing the transcription factors Oct3/4, Sox2, Klf4, and c-Myc (Takahashi and Yamanaka, 2006). Pluripotent colonies appeared two to three weeks after infection and were kept on irradiated mouse embryonic fibroblasts. After several passages, the iPS cells were transferred on Matrigel, but spontaneous differentiation occurred on this feeder-less system (**Figure 2A**). Further investigations showed that a higher concentration of basic fibroblast growth factor (bFGF) was essential to maintain these cynomolgus cells in an undifferentiated state (**Figure 2B**).

### 3.2 Characterization of the iPS cells from *Macaca fascicularis*

**Figure 3:**
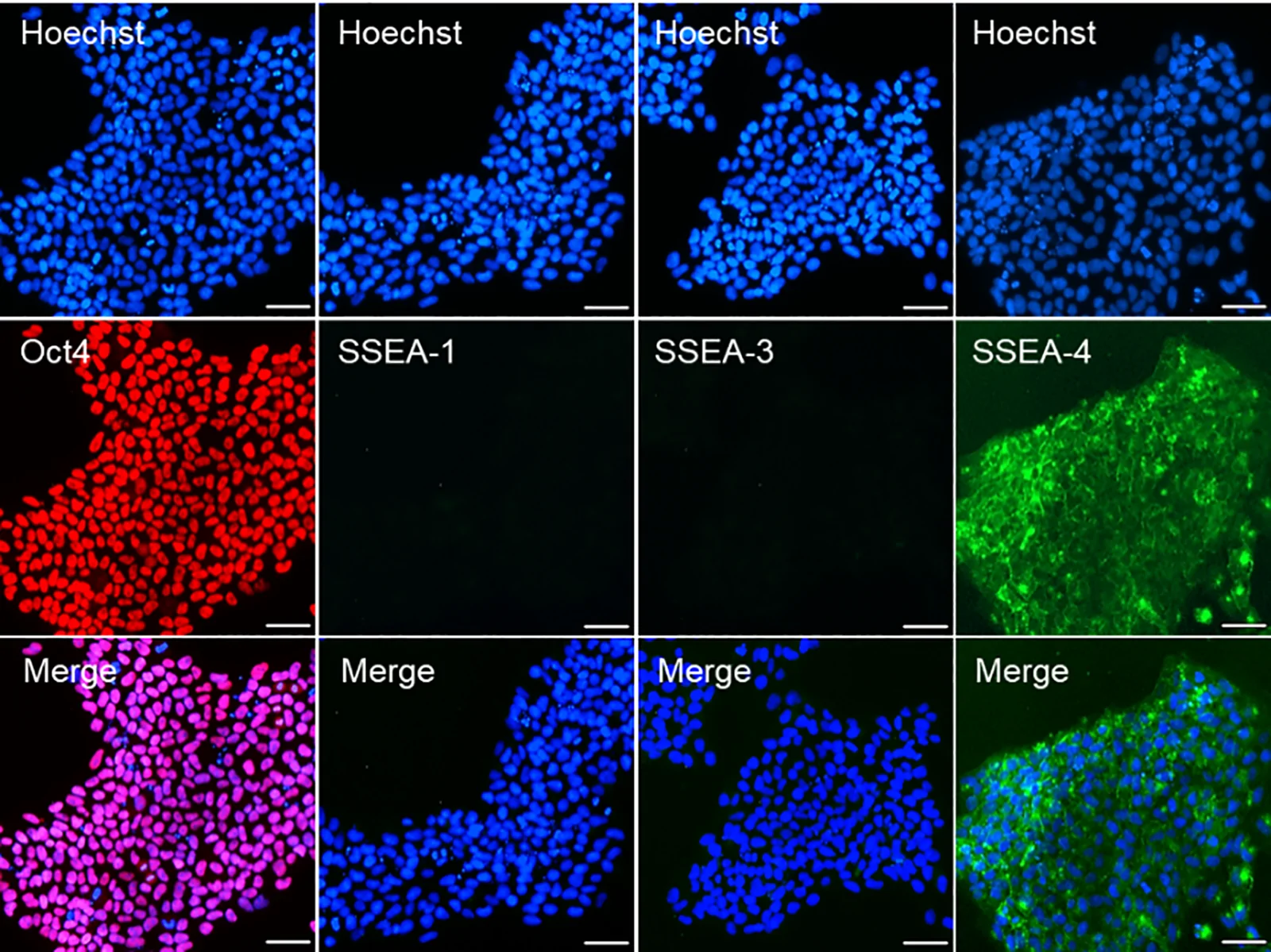
Selected clone #13 from monkey PDF091812. Immunofluorescence analysis of Oct4 (red), and stage-specific embryonic antigens (SSEA-1, SSEA-3, and SSEA-4) (green) in cynIPS cells. Nuclei were counterstained with Hoechst (blue). Scale bars, 50 µm.

**Figure 4:**
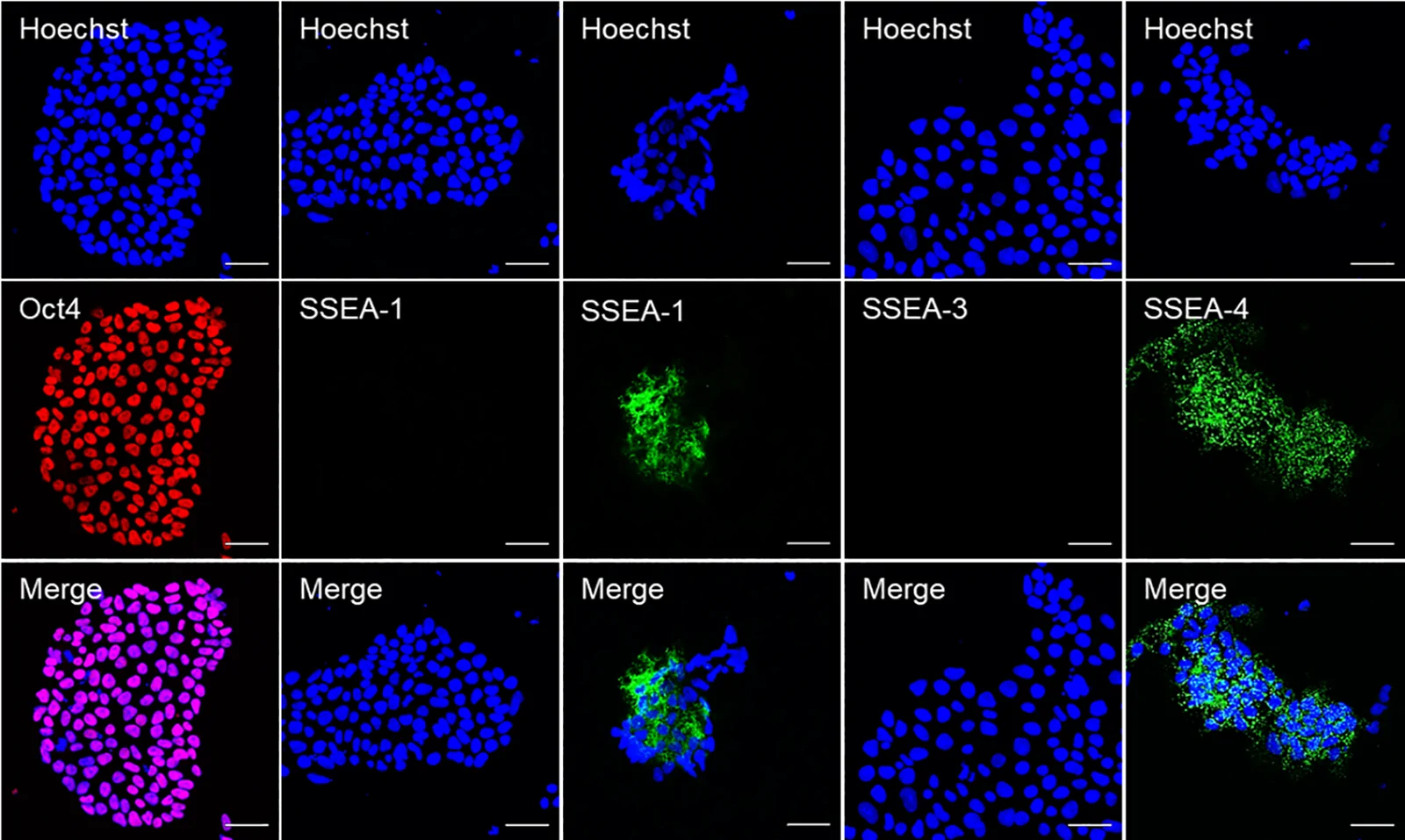
Example of a clone not selected based on SSEA-1 expression. Immunofluorescence analysis of an iPS clone from monkey 5501 of Oct4 (red), SSEA-1, SSEA-3, and SSEA-4 (green). Nuclei were counterstained with Hoechst (blue). Scale bars, 50 µm.

**Figure 5:**
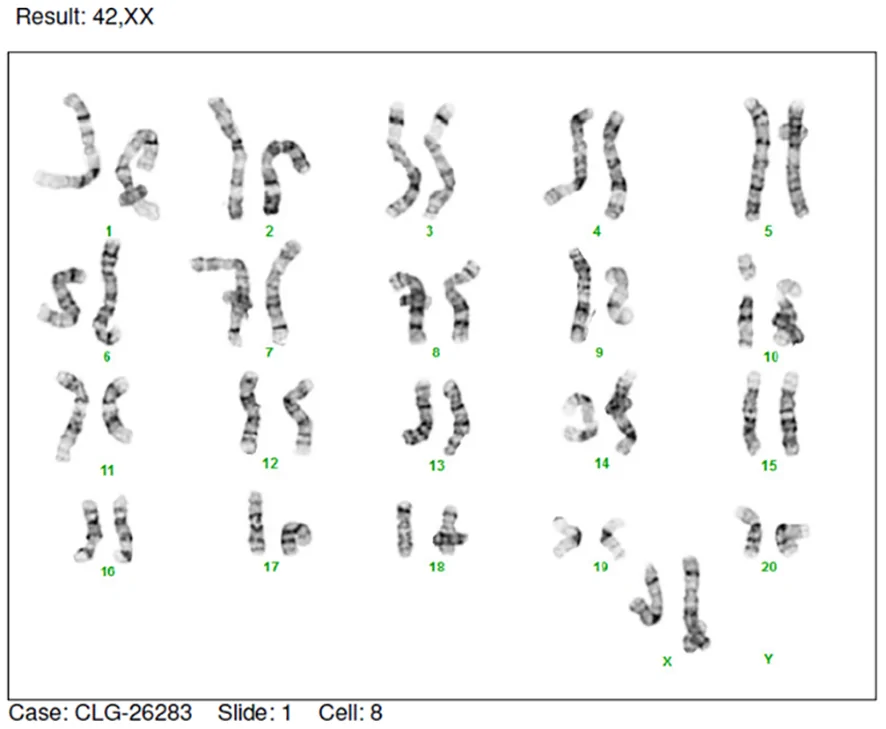
Karyotype analysis of cynIPS cells from clone #13 by array Comparative Genomic Hybridization method.

**Figure 6:**
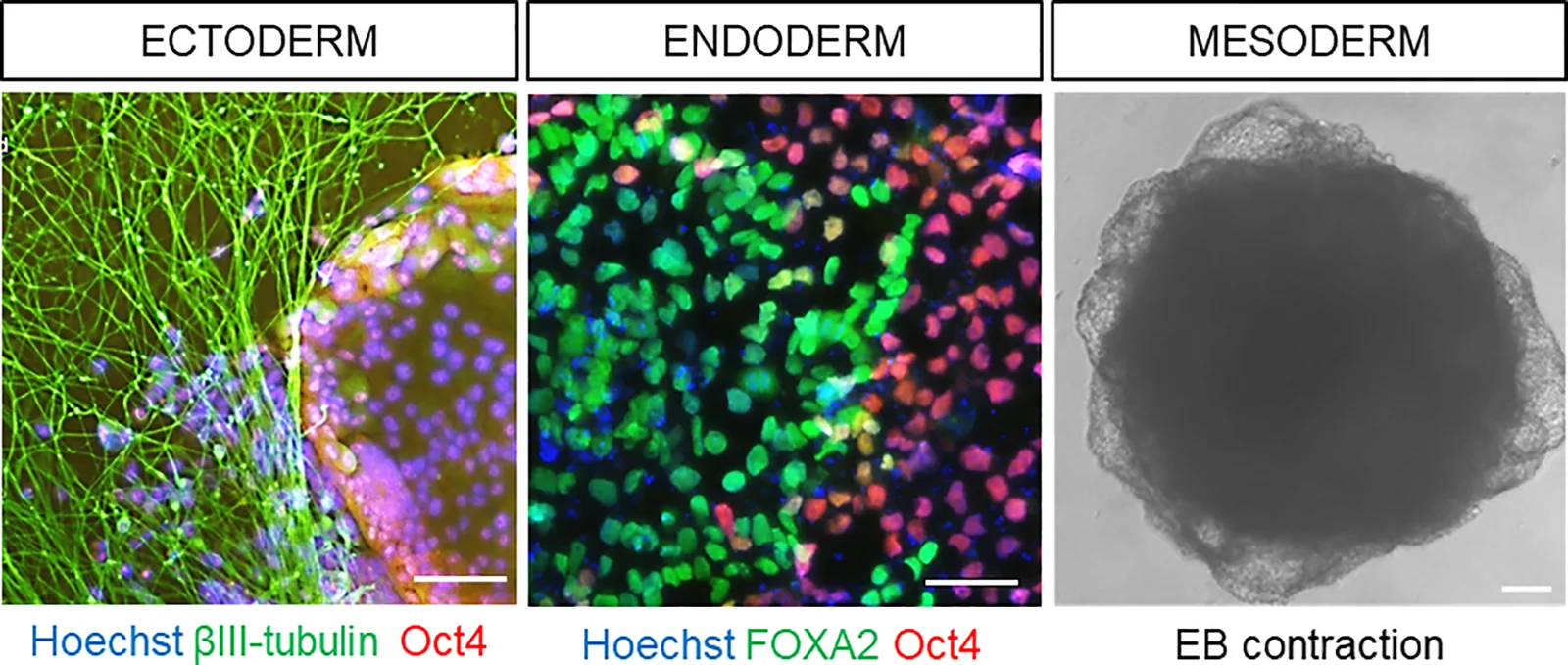
Differentiation potential of cynIPS cells from clone #13. Upon stimulation, cells expressed βIII-tubulin (green, ectoderm marker), FOXA2 (green, endoderm marker) and contracted spontaneously after EB formation (mesoderm). Non-responsive cells remained pluripotent and expressed Oct4 (red). Nuclei were counterstained with Hoechst (blue). Scale bars, 100 µm.

Following the clonal expansion phase, clones exhibiting typical human embryonic stem cell-like morphology (flat and tightly packed colonies of round cells with large nuclei) were selected and fully characterized by immunofluorescence (**Figures 3**, **4**). Based on these results, one clone (#13) was selected and checked for karyotype (**Figure 5**), and differentiation potential into the three germ layers (**Figure 6**).

### 3.3 Generation of hepatocyte-like cells from cynomolgus iPS cells

**Figure 7:**
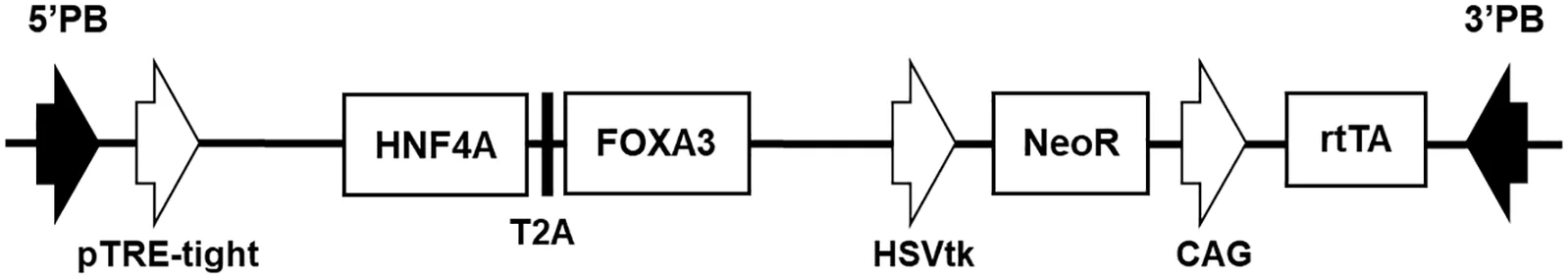
Schematic representation of the Dox-inducible construct. PB, PiggyBac DNA transposon; TRE, TET responsive element; HNF4A, Coding sequence of hepatocyte nuclear factor 4 alpha; FOXA3, Coding sequence forkhead box protein A3); T2A, self-cleavage peptides from *Thosea asigna* virus; HSVtk, Minimal promoter fragment from the HSV thymidine kinase promoter; NeoR, neomycin resistance gene; CAG, chicken β-actin promoter; rtTA, TET transactivator protein gene.

The generation of human hepatocytes from stem cells is a well-known process and diverse protocols have been developed (Si-Tayeb et al., 2010; Chen et al., 2012; Gao et al., 2017; Kotaka et al., 2017; Takeishi et al., 2020; Tomaz et al., 2022), but the translation of this process to cynomolgus cells required extensive tailoring. The stepwise approach, which consists of adding growth factors mimicking liver embryogenesis, was not robust and reproducible enough (data not shown). Therefore, we used a direct differentiation approach to generate cynomolgus monkey hepatocyte-like cells (cynHLCs) by overexpressing essential transcription factors for hepatic lineage formation (Huang et al., 2014). Using an inducible construct based on a tetracycline ON/OFF system (**Figure 7**), hepatocyte nuclear factor 4 homeobox A (HNF4A) and forkhead box protein A3 (FOXA3) were overexpressed in cynIPS cells to drive endoderm formation and hepatocyte maturation (Kang et al., 2018; Yoon et al., 2018).

**Figure 8:**
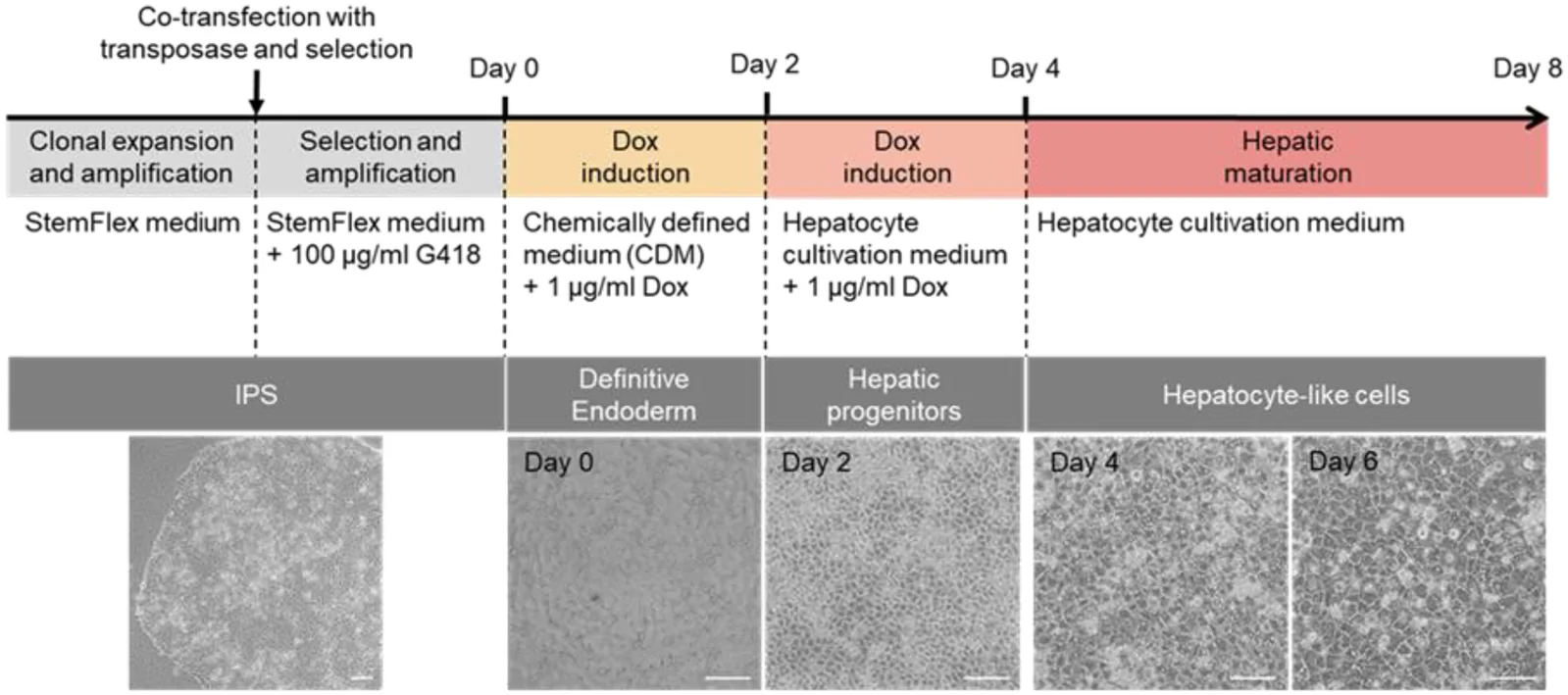
CynHLCs generation from iPS cells. Schematic representation of the differentiation protocol and brightfield images of differentiating cynIPS cells at the indicated time points. G418: geneticin; Dox: doxycycline. Scale bars, 100 µm.

**Figure 9:**
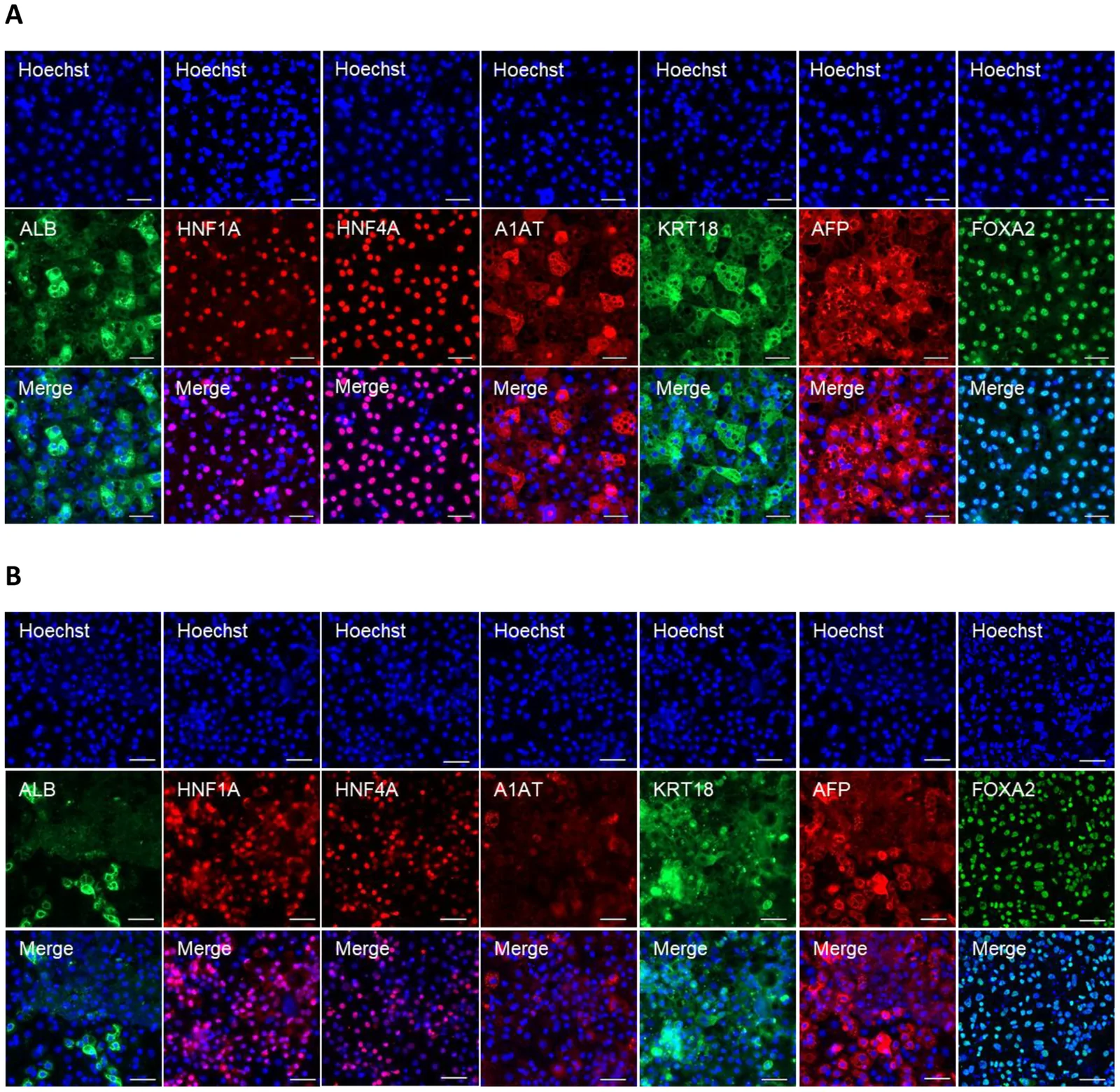
Immunofluorescence of cynHLCs at day 6 of selected clone #13.5 **(A)** and clone #13.6 **(B)** with antibodies to HNF1A, HNF4A, AFP, A1AT (red), ALB, KRT18, and FOXA2 (green). Nuclei were visualized with Hoechst (blue). Scale bars, 50 μm.

**Figure 10:**
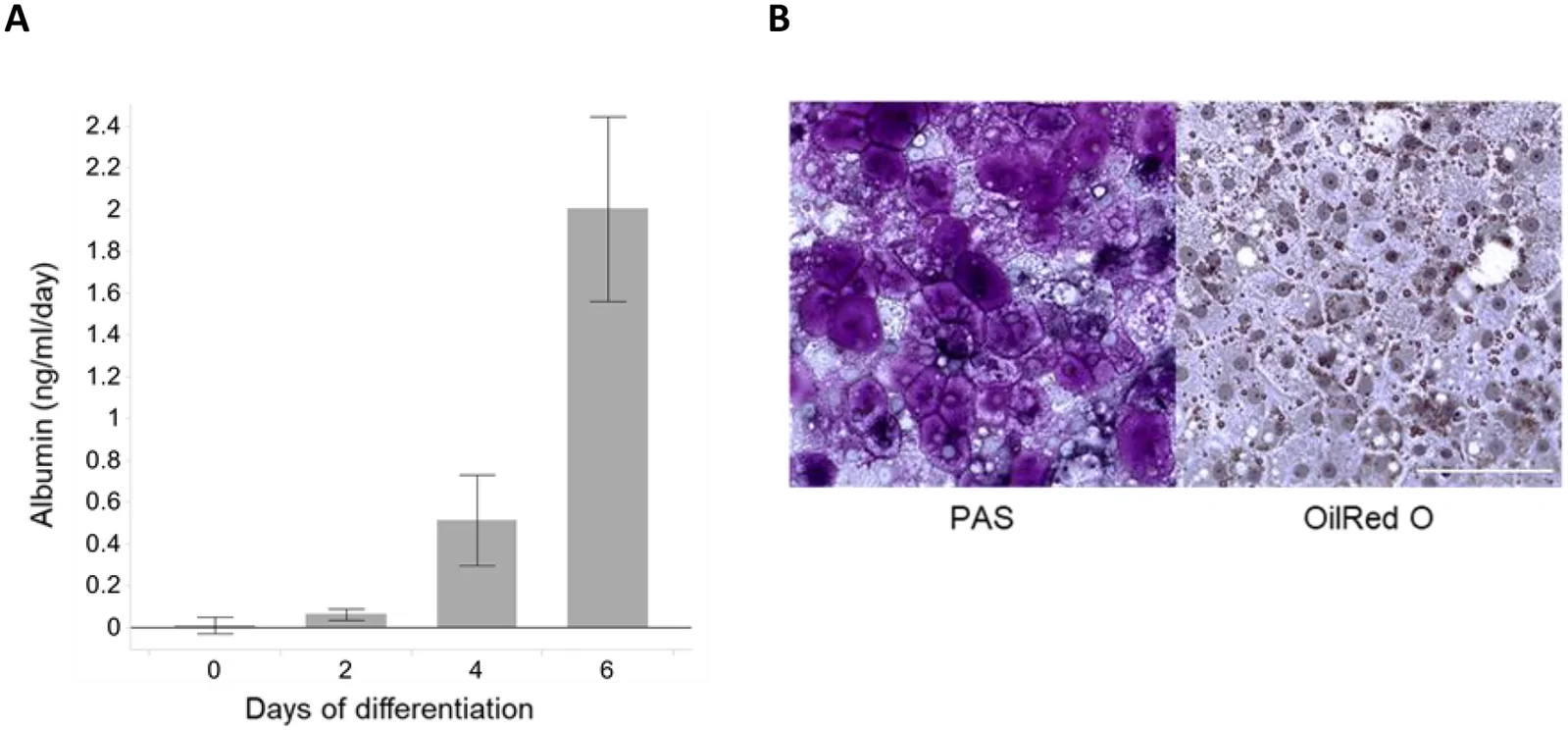
**(A)** Enzyme-linked immunosorbent assay (ELISA) for ALB in the supernatants at indicated time points. n=3. **(B)** Periodic Acid Schiff (PAS) and Oil Red O staining of cynHLCs after 10 days of differentiation. Scale bar, 100 μm.

Briefly, a confluent pluripotent cell population was induced for 4 days with doxycycline (Dox), and hepatic progenitor cells were further matured for 2 days to generate cynHLCs (**Figure 8**). CynHLCs produced from clone HNF4A/FOXA3-#13.5 displayed the best hepatic characteristics, such as expression of the genes for albumin (ALB), hepatocyte nuclear factor 1 homeobox A (HNF1A), HNF4A, α1-antitrypsin (A1AT), cytokeratin 18 (KRT18), α-fetoprotein (AFP), and forkhead box A2 (FOXA2) (**Figures 9A, B**) and increasing levels of albumin secretion (**Figure 10A**). Additional characterization methods revealed accumulation of lipid droplets (Oil Red O staining) and glycogen storage (Periodic Acid Schiff staining), which are attributes of hepatocytes (**Figure 10B**).

### 3.4 Characterization of the cynomolgus hepatocyte-like cells

**Figure 11:**
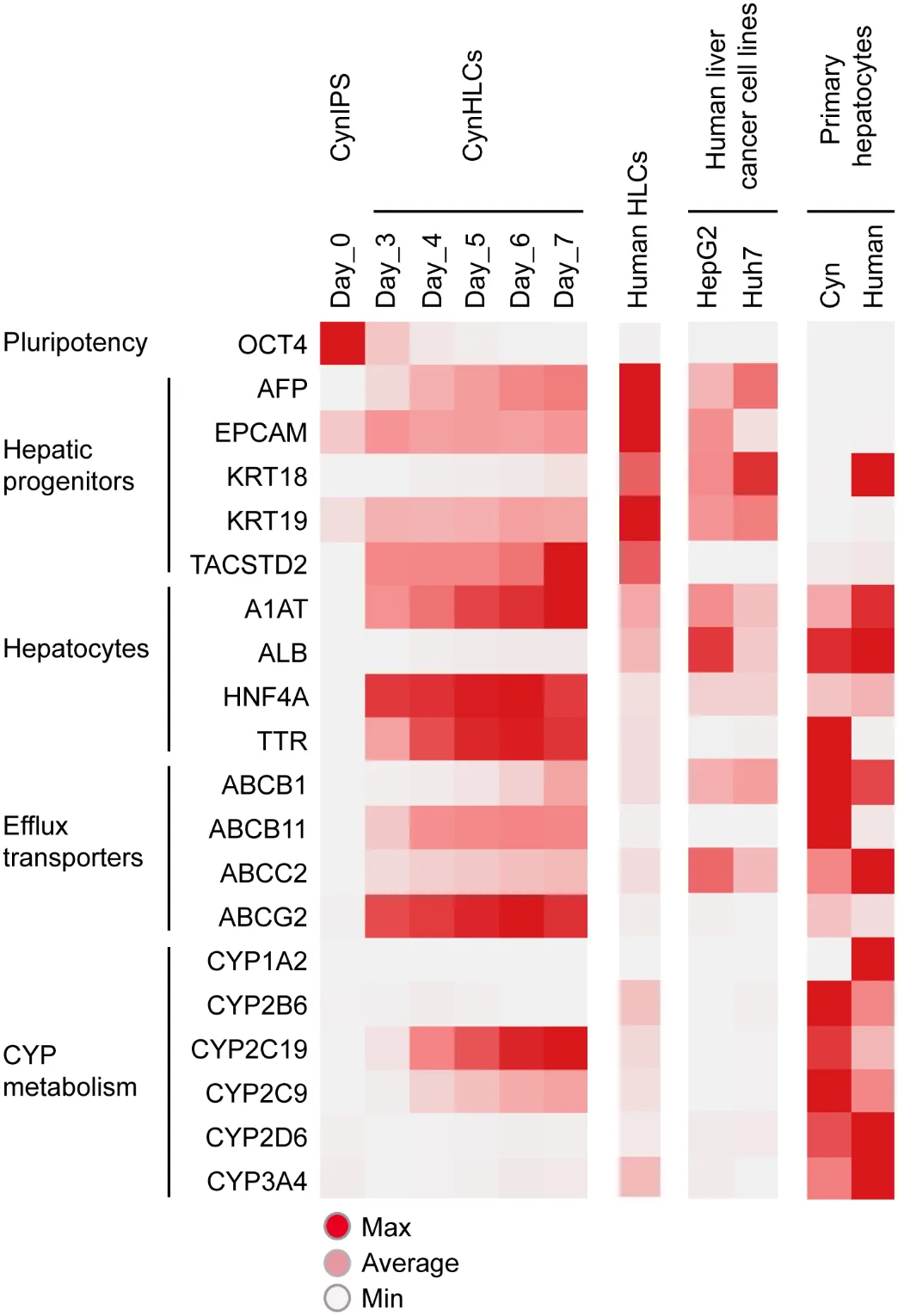
Heatmap of the RNA-seq based mean expression of cynIPS cells (Day_0), cynHLCs (Day_3 to Day_7), human HLCs, human liver cancer cell lines (HepG2 and Huh7), and primary hepatocytes for selected genes indicative of cell types (pluripotency, hepatic progenitors, and hepatocytes) or cellular processes (efflux transporters and CYP metabolism). Expression values in transcripts per million (tpm) are colored relative to the maximum (red) and minimum (white) expression value for each gene.

**Figure 12:**
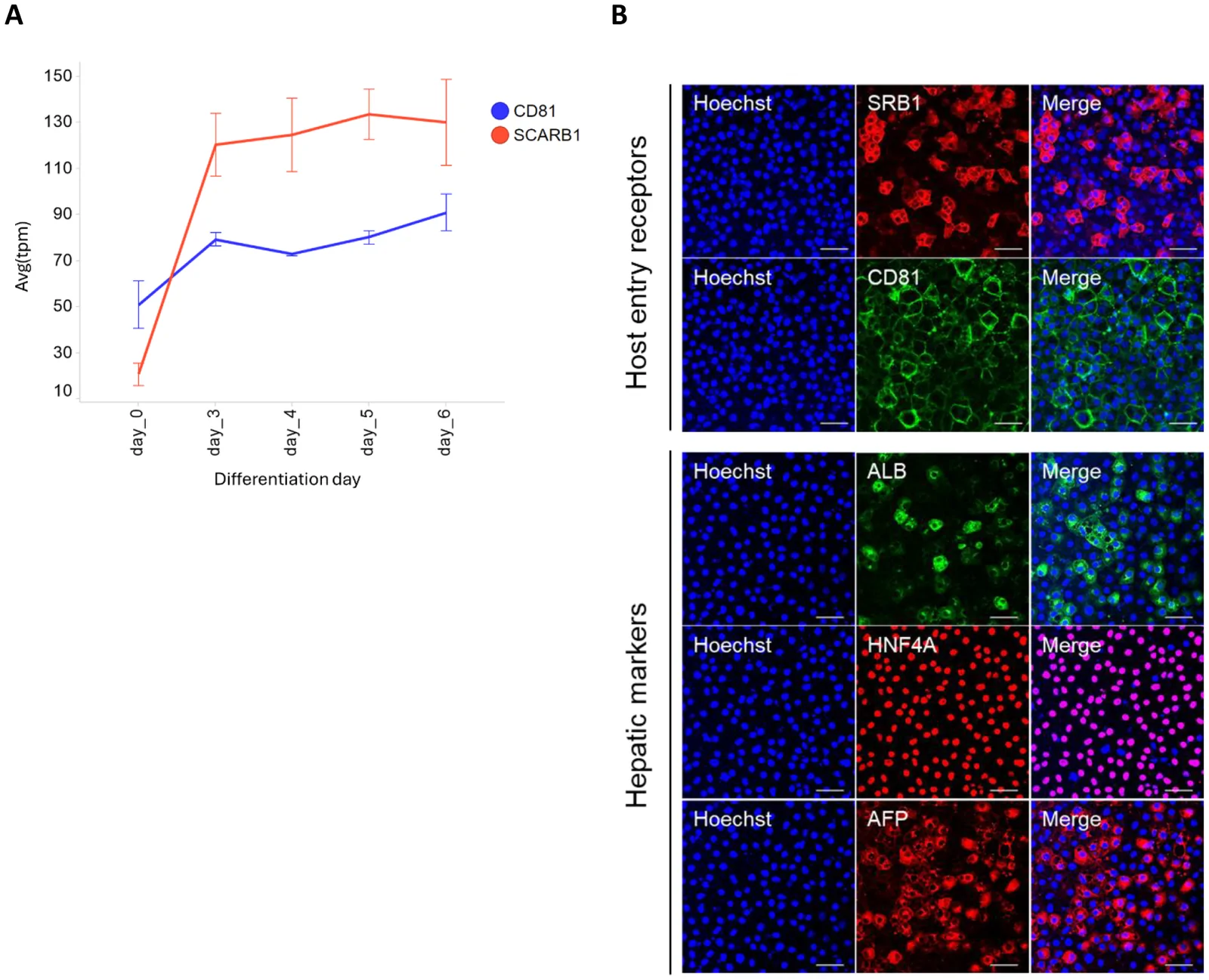
Hepatic signature and expression of host entry receptors indicate permissiveness of cynHLCs to *Plasmodium* parasite. **(A)** Bar charts representing mRNA levels in tpm of SRB1 (SCARB1) and CD81 at day 0, 3, 4, 5, and 6. Error bars represent standard deviation. n=3. **(B)** Immunofluorescence of cynHLCs at day 4 with antibodies to the hepatic markers ALB (green), HNF4A (red), and AFP (red) and to the host entry receptors SRB1 (red) and CD81 (green). Nuclei were visualized with Hoechst (blue). Scale bars, 50 μm.

RNA-seq analysis of cynHLCs confirmed the hepatic signature of the generated cells (**Figure 11**). Comparison with human HLCs as well as liver cancer cell lines (HepG2 and Huh7) showed a very high similarity on the gene expression profile. While differentiating, the obtained cynHLCs lost expression of pluripotent markers and expressed typical hepatic progenitor genes and mature hepatic genes including efflux transporters at day 7. We also assessed the expression of known *Plasmodium* host entry receptors such as cluster of differentiation 81 (CD81) and scavenger receptor class B type 1 (SRB1) (Rodrigues et al., 2008; Yalaoui et al., 2008a, b; Manzoni et al., 2017) in the generated cynHLCs. Expression of SRB1 and CD81 was confirmed in cynHLCs at the mRNA level during the differentiation process (**Figure 12A**). CD81 was gradually expressed in iPS (day 0) and differentiating cells. In contrast, SRB1 was weakly expressed in iPS cells, but highly and constantly after day 3, suggesting that cynHLCs become permissive to *Plasmodium* only when they acquire a hepatic signature (Ng et al., 2015). CynHLCs were infected after 4 days of differentiation, when they expressed hallmark hepatic markers like ALB, HNF4A, and AFP in addition to the host entry receptors SRB1 and CD81 (**Figure 12B**). Although SRB1 was already highly expressed at day 3, *P. cynomolgi* sporozoite inoculation was performed at day 4 to avoid potential parasite inhibition due to doxycycline (Pang et al., 1988; Gaillard et al., 2015).

### 3.5 Maintenance of the cynomolgus hepatocyte-like cells

**Figure 13:**
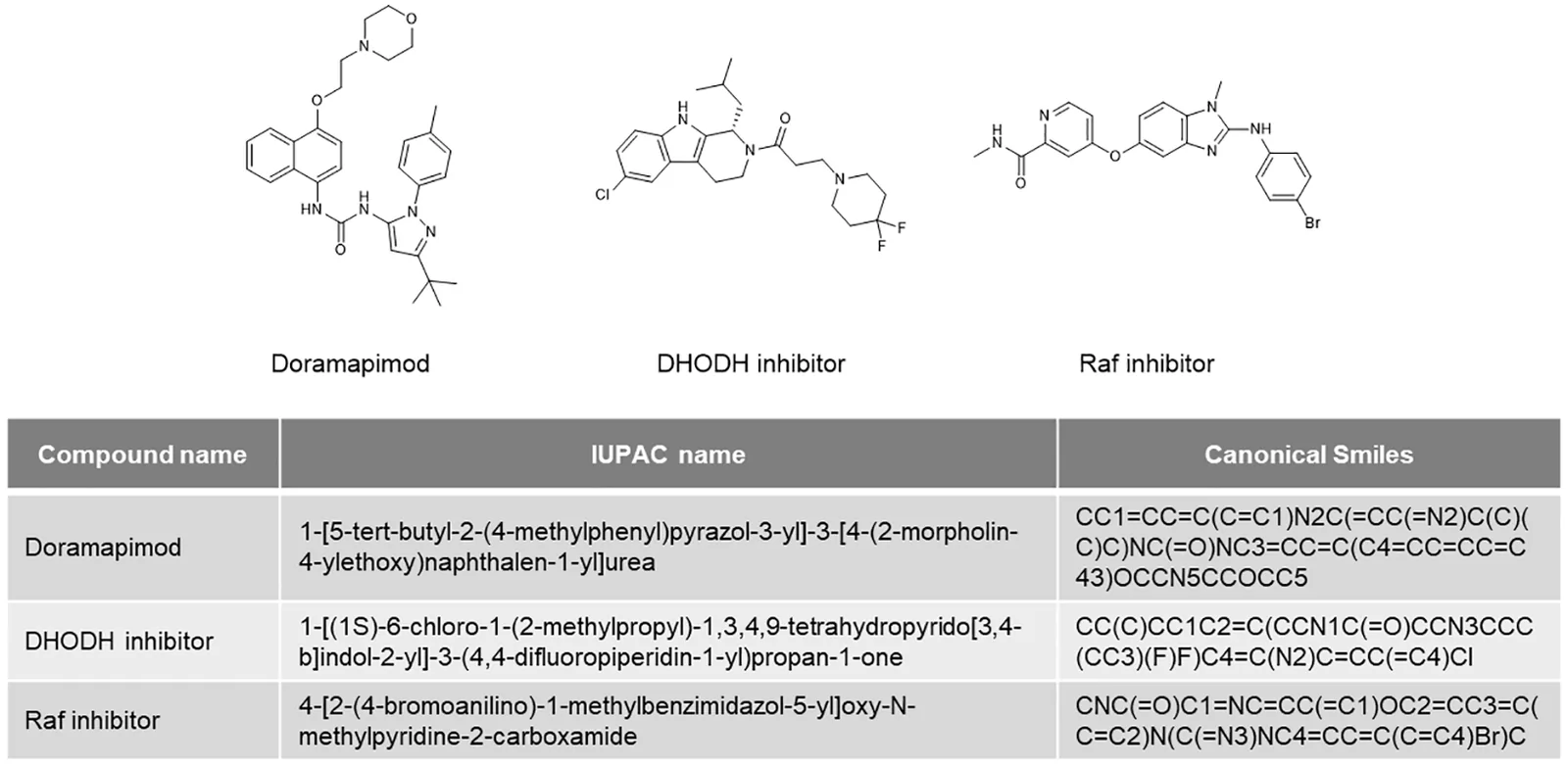
Chemical structures of the hit compounds identified in survival screen (doramapimod, DHODH inhibitor, and Raf inhibitor) and table displaying the International Union of Pure and Applied Chemistry (IUPAC) names and the canonical smiles of each compound.

While the differentiation process was extremely fast with the cynomolgus cells, the maintenance of the hepatic stage was challenging. After 8 days of differentiation, cynHLCs started to detach and died. In order to improve cell survival, we conducted a screen against an in-house chemical library of well-characterized compounds. Three hit compounds that improved cell viability were identified: a stress-activated protein kinase 2/p38β (SAPK2/p38β) inhibitor named doramapimod, a dihydroorotate dehydrogenase (DHODH) inhibitor, and a rapidly accelerated fibrosarcoma (Raf) inhibitor (**Figure 13**).

**Figure 14:**
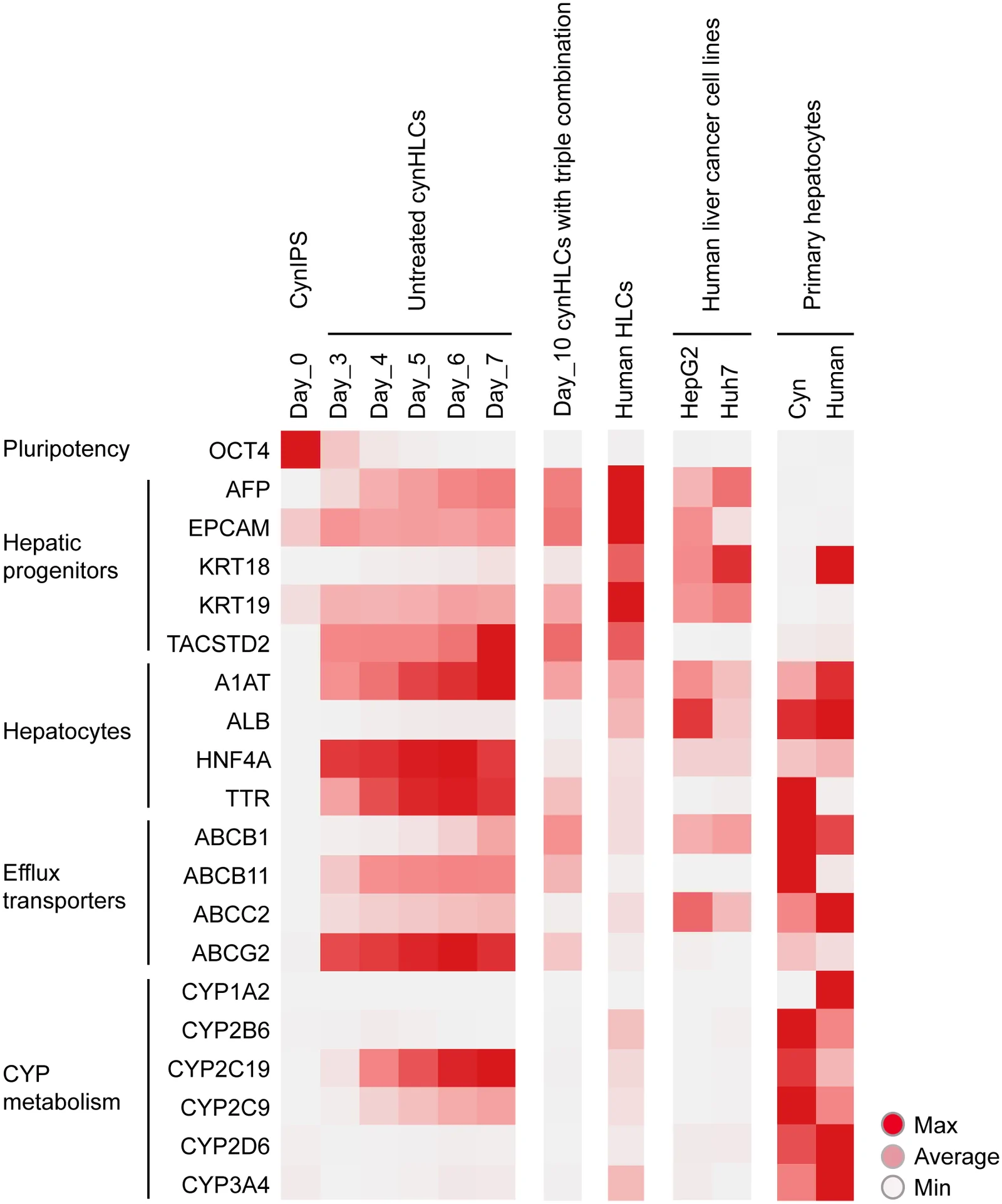
Heatmap of the RNA-seq based mean expression of cynIPS cells (Day_0), cynHLCs (Day_7 untreated and Day_10 triple combination), and human HLCs for selected genes indicative of cell types (pluripotency, hepatic progenitors, and hepatocytes) or cellular processes (efflux transporters and CYP metabolism). Expression values in transcripts per million (tpm) are colored relative to the maximum (red) and minimum (white) expression value for each gene.

Daily treatment with a mix containing the three hits prolonged hepatocyte survival up to 17 days of differentiation (**Figure 1A**) and maintained ALB and HNF4 expression (**Figure 1B**). With the triple combination, cell viability remained constant from day 6 to day 12 whereas it dramatically decreased when cells were not treated (DMSO control) or treated with only one hit compound (**Figure 1C**). Although the treated cynHLCs showed a more precursor-like mRNA signature than untreated cynHLCs (**Figure 14**), the triple combination finally allowed to study liver stage parasite development for at least 12 days, a timeframe necessary to investigate the dormant stage of malaria.

### 3.6 Infection of the cynomolgus hepatocyte-like cells with *Plasmodium cynomolgi*

**Figure 15:**
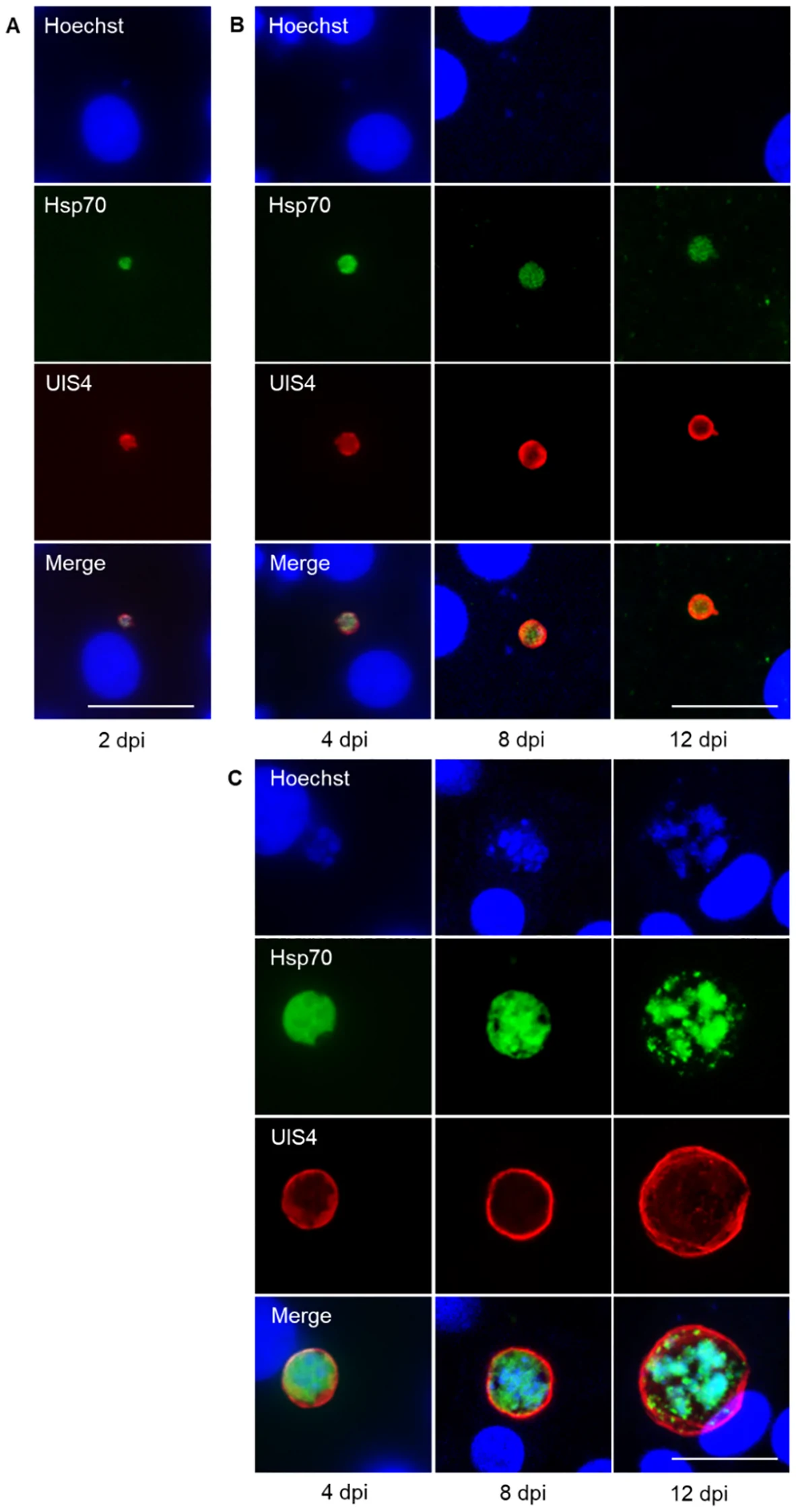
Infection of cynHLCs with *P. cynomolgi* sporozoites. Immunofluorescence of liver stage parasites with antibodies specific for *P. cynomolgi*-Hsp70 (green) and *P. cynomolgi*-UIS4 (red) at 2 dpi **(A)**, 4–12 dpi small forms **(B)** and 4–12 dpi large forms **(C)**. Cell nuclei and parasite DNA were visualized with Hoechst (blue). Scale bars, 20 µm.

**Figure 16:**
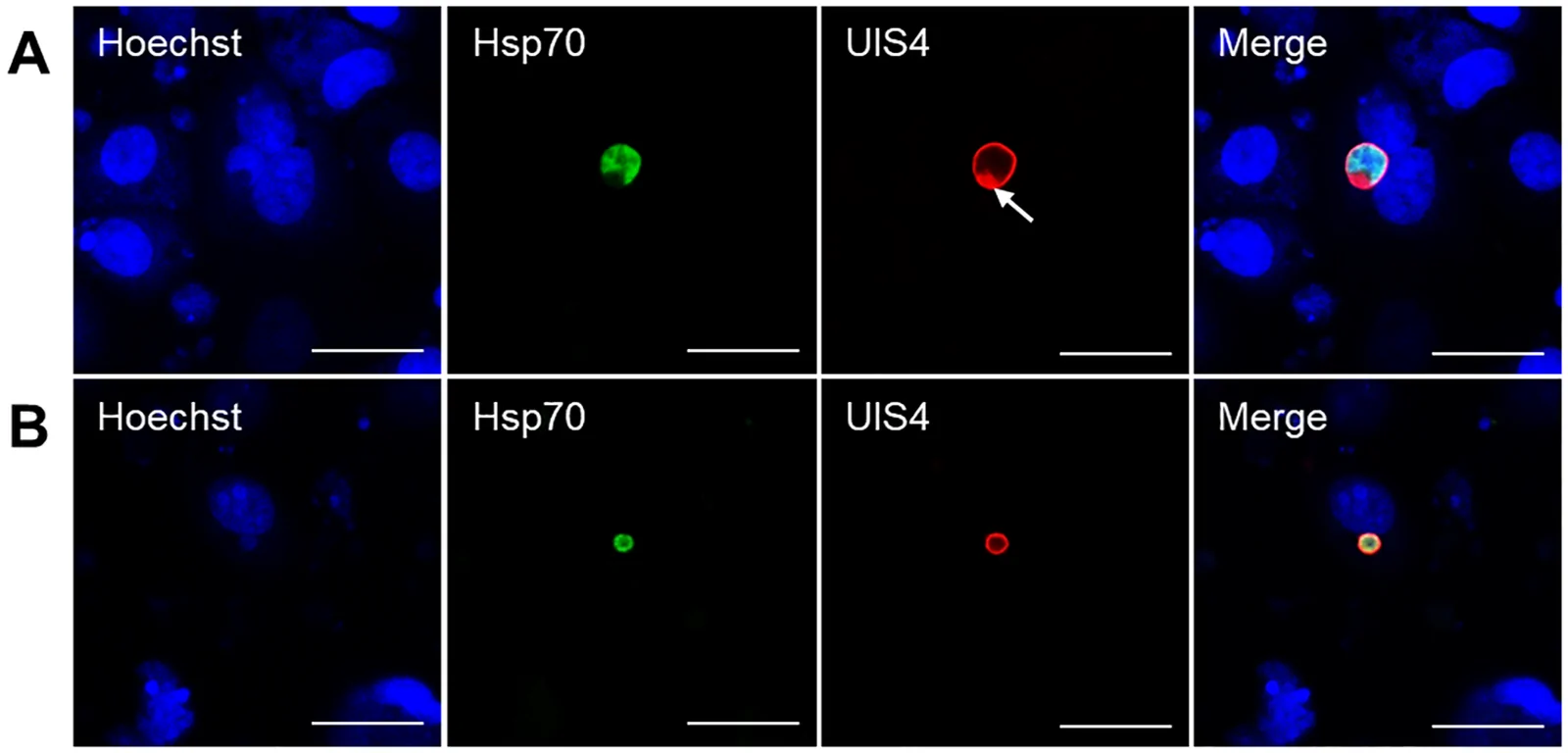
Immunofluorescence of liver-stage parasites **(A)** large form and **(B)** small form. Prominence (white arrow) observed in a subset of schizonts. All liver stage parasites were visualized with antibodies specific for *P. cynomolgi* Hsp70 (green) and UIS4 (red). Host cell nuclei and parasite DNA content were visualized with Hoechst (blue). Scale bars, 20 µm.

Infection of 4-days differentiated cynHLCs was performed with freshly isolated *P. cynomolgi* sporozoites from *Anopheles stephensi* mosquitoes. We detected the first liver stage parasites at 2 days post-infection (dpi) after fixation and staining with *P. cynomolgi* anti-Hsp70 (70 kilodalton heat shock proteins) antibodies. An additional staining with antibodies against UIS4 (up regulated in infective sporozoites gene 4) confirmed that the parasitophorous vacuole membrane (PVM) of the liver stage parasites had been formed (Bertschi et al., 2018). At 2 dpi, we observed a uniform population of uninucleated parasites with a size of 3 µm (**Figure 15A**). Despite the low infection rate of less than 10 parasites per well in a 96-well plate, two populations of liver stage parasites were clearly distinguishable by 4 dpi. The first (**Figure 15B**) was composed of stationary parasites that stayed uninucleated over time (Dembele et al., 2014). The second population (**Figure 15C**) was composed of growing and multinucleated forms, which would lead to the formation of liver schizonts. It is currently unclear if a PVM prominence detected by anti-UIS4 antibodies would be a reliable hypnozoite-specific marker (Mikolajczak et al., 2015; Gural et al., 2018; Schafer et al., 2018). In our system, PVM prominence was found in a subset of developing liver stage parasites (**Figure 16**), which would suggest that PVM prominence is not sufficient to discriminate between dormant and developing liver stage parasites. Therefore, in the absence of a *P. cynomolgi* hypnozoite-specific marker, we defined a hypnozoite as a small, round liver stage parasite (diameter <8 µm) containing a single nucleus. In comparison, a developing schizont is bigger in size and contains at least two nuclei. Small and uninucleated forms were observed until 12 dpi. As previously described (Mikolajczak et al., 2015), the dormant parasites were slightly increasing in size (from 3 µm at 4 dpi to 5 µm at 12 dpi), but the single nucleus observed by Hoechst staining confirmed the dormant state of these liver stage parasites. The current assay conditions did not allow us to visualize hypnozoite reactivation and subsequent development into mature liver schizonts as described for *P. cynomolgi* in primary cynomolgus monkey hepatocytes (Dembele et al., 2014).

## 4 Discussion

We report here for the first time the generation of cynHLCs from *M. fascicularis*, the natural host of *P. cynomolgi*. These cynHLCs can be generated *in vitro* at high numbers, and they exhibit the hallmarks of hepatocytes in terms of morphology, protein production, and gene expression. Thus, they provide a useful alternative to primary human or simian hepatocytes for research purposes, and they are preferable to primary cells in terms of reproducibility and ethics. Furthermore, the cynHLCs are genetically amenable at the iPS stage, i.e. before differentiation to hepatocyte-like cells. As host cells for malaria parasites, the cynHLCs provide a new system to study host-pathogen interaction and the biology of *Plasmodium* liver stages. These include the most elusive form of the malaria parasites, the hypnozoite, which is produced by species such as *P. vivax* or *P. cynomolgi*. A better understanding of the hypnozoites will be necessary for the successful elimination of malaria (Radha et al., 2025).

Several hurdles had to be overcome to develop the cynHLC *in vitro* system. Key to the cultivation of *M. fascicularis* iPS cells was the finding that basic fibroblast growth factor (bFGF) needs to be present throughout *in vitro* culture to maintain the iPS cells in an undifferentiated state. We used commercially available human bFGF; the human and cynomolgus bFGF orthologs (GenBank entries NP_001348594 and XP_045247784, respectively) are 100% identical at the amino acid level. The differentiation of the cynIPS cells to cynHLCs was challenging as well, as the simple addition of growth factors for liver embryogenesis to the medium did not yield the desired result. The successful differentiation of cynIPS cells towards hepatocytes was achieved with a genetic approach, by inducible expression of the transcription factors HNF4A and FOXA3. The final obstacle was the short lifespan of the generated cynHLCs in culture. This problem was solved with a phenotypic screen for small molecules that improve cynHLC viability, which returned three hits: a MAPK inhibitor, Raf inhibitor, and DHODH inhibitor. This is in agreement with the concept that the inhibition of the MAPK pathway slows down cell proliferation and differentiation, and that the pathway is linked to pyrimidine *de novo* synthesis (Koundinya et al., 2018; Wang et al., 2019). A cocktail of the three identified inhibitors prolonged the lifespan of the cynHLC, which allowed to proceed and infect them with malaria parasites.

Proof-of-concept was obtained for the successful infection of the generated cynHLC with *P. cynomolgi* sporozoites isolated from the salivary glands of infected female *A. stephensi* mosquitoes. By day 2 after infection, a uniform population of intracellular parasites residing in a parasitophorous vacuole had established, which by day 4 further developed into two distinct populations: large multinuclear vs. small mononuclear forms, representing *P. cynomolgi* liver schizonts and hypnozoites, respectively. Although we are lacking formal proof of hypnozoites in the sense that they can be woken up and transformed to schizonts, the fact that the small forms stayed mononuclear for the remaining observation time, i.e. until day 12, supports the notion that they can serve as models for hypnozoites. Thus, we think that the results achieved with this new infection model warrant further development. It will be critical to improve the infection rate, in particular for drug efficacy testing against hypnozoites. Furthermore, combining the iPS technology with *in vitro* cultured *P. cynomolgi* erythrocytic stages (Chua et al., 2019a) would make the *P. cynomolgi* system more accessible and more importantly, largely independent of primates. In conclusion, this simian iPS-based *in vitro* system provides a promising alternative to *P. vivax in vitro* work to elucidate the underlying mechanisms of relapsing malaria.

## Data Availability

The transcriptomics data are available from the NCBI Sequence Read Archive (www.ncbi.nlm.nih.gov/sra/) under accession number SRP256529.

## Supplementary material

Supplementary Table 1 Primary and secondary antibodies used for immunofluorescence analysis.
