# Supplementary material for: Simian iPS-derived hepatocytes support Plasmodium cynomolgi infection and hypnozoite formation
Source: Front Cell Infect Microbiol. 2026 Aug 14;16:1871289. doi: 10.3389/fcimb.2026.1871289 (PMC13522909; doi:10.3389/fcimb.2026.1871289)
Supplement: Supplementary file 1 [file DataSheet1.pdf]

Simian iPS-derived hepatocytes support *Plasmodium cynomolgi* infection and hypnozoite formation

Mélanie Pellisson[1,2,3], Anne-Marie Zeeman[4], Thierry Doll[3], Lucy Kirchhofer-Allan[5], Sven Schuierer[3], Guglielmo Roma[3], Erika L. Flannery[5], Sebastian Mikolajczak[5], Clemens H. M. Kocken[4], Pascal Mäser[1,2*], Matthias Rottmann[1,2], Matthias Müller[3*]

[1]Swiss Tropical and Public Health Institute (Swiss TPH), Allschwil, Switzerland.

[2]University of Basel, Basel, Switzerland.

[3]Novartis Institutes for Biomedical Research, Novartis Pharma AG, Basel, Switzerland.

[4]Biomedical Primate Research Centre, Department of Parasitology, Rijswijk, The Netherlands.

[5]Novartis Institute for Tropical Diseases, Novartis Pharma AG, Emeryville, United States.

Supplementary Material

Name	Symbol	Provider	Reference	Specie	Dilution
Alpha-1-antitrypsin	A1AT	Dako	A0012	Rabbit	1:10,000
Alpha-1-Fetoprotein	AFP	Dako	A0008	Rabbit	1:500
Albumin	ALB	Bethyl	A80-129A	Goat	1:500
Class III β-tubulin	βIII-tubulin	Sigma	T8660	Mouse	1:5'000
Cluster of Differentiation 81	CD81	Invitrogen	MA5-13548	Mouse	1:100
Forkhead box protein A2	FOXA2	Abcam	ab60721	Mouse	1:50
Cytokeratin 18	KRT18	Abcam	ab82254	Mouse	1:100
Hepatocyte nuclear factor 1A	HNF1A	Cell Signaling Technologies	12425	Rabbit	1:100
Hepatocyte nuclear factor 4A	HNF4A	Cell Signaling Technologies	3113	Rabbit	1:100
Octamer-4	Oct4	Stemgent	09-0023	Rabbit	1:1,000
P. cynomolgi 70 kilodalton heat shock proteins	Pc-Hsp70	Anne-Marie Zeeman, BPRC	20285	Rabbit	1:1,000
P. cynomolgi up-regulated in infective sporozoites gene 4	Pc-UIS4	Anne-Marie Zeeman, BPRC	5262527	Rat	1:400
Scavenger Receptor class B member 1	SRB1	Novus Biologicals	NB400-104	Rabbit	1:100
Stage-specific embryonic antigen-1	SSEA-1	Chemicon/Milipore	MAB4301	Mouse	1:200
Stage-specific embryonic antigen-3	SSEA-3	Stemgent	09-0014	Rat	1:200
Stage-specific embryonic antigen-4	SSEA-4	Stemgent	09-0006	Mouse	1:1,000
Alexa Fluor 555 donkey anti-rabbit	Dk@Rb	Invitrogen	A31572	Donkey	1:1,000
Alexa Fluor 488 donkey anti-goat	Dk@Gt	Invitrogen	A11055	Donkey	1:1,000
Alexa Fluor 488 goat anti-mouse	Gt@Ms	Invitrogen	A10667	Goat	1:1,000
Alexa Fluor 594 chicken anti-rat	Ch@Rt	Invitrogen	A21471	Chicken	1:1,000
Goat anti-rabbit IgG-FITC	Gt@Rb	Anne-Marie Zeeman, BPRC		Goat	1:200
Hoechst	Hoechst	Invitrogen	33342	N.A.	1:10,000

Table S1. Primary and secondary antibodies used for immunofluorescence analysis.
